# A versatile cell-penetrating peptide-adaptor system for efficient delivery of molecular cargos to subcellular destinations

**DOI:** 10.1371/journal.pone.0178648

**Published:** 2017-05-26

**Authors:** Verra M. Ngwa, David S. Axford, Allison N. Healey, Scott J. Nowak, Carol A. Chrestensen, Jonathan L. McMurry

**Affiliations:** 1 Department of Chemistry & Biochemistry, Kennesaw State University, Kennesaw, Georgia, United States of America; 2 Department of Molecular & Cellular Biology, Kennesaw State University, Kennesaw, Georgia, United States of America; 3 New Echota Biotechnology, Kennesaw, Georgia, United States of America; Helsingin Yliopisto, FINLAND

## Abstract

Cell penetrating peptides have long held great potential for delivery of biomolecular cargos for research, therapeutic and diagnostic purposes. They allow rapid, relatively nontoxic passage of a wide variety of biomolecules through the plasma membranes of living cells. However, CPP-based research tools and therapeutics have been stymied by poor efficiency in release from endosomes and a great deal of effort has been made to solve this ‘endosomal escape problem.’ Previously, we showed that use of a reversible, noncovalent coupling between CPP and cargo using calmodulin and a calmodulin binding motif allowed efficient delivery of cargo proteins to the cytoplasm in baby hamster kidney and other mammalian cell lines. The present report demonstrates the efficacy of our CPP-adaptor scheme for efficient delivery of model cargos to the cytoplasm using a variety of CPPs and adaptors. Effective overcoming of the endosomal escape problem is further demonstrated by the delivery of cargo to the nucleus, endoplasmic reticulum and peroxisomes by addition of appropriate subcellular localization signals to the cargos. CPP-adaptors were also used to deliver cargo to myotubes, demonstrating the feasibility of the system as an alternative to transfection for the manipulation of hard-to-transfect cells.

## Introduction

Cell-penetrating peptides (CPPs, also known as protein transduction domains, or PTDs) allow transport of biomolecular cargo into an array of eukaryotic cells. Discovered some decades ago, there has been a great deal of interest ever since, including development of an array of CPP moieties [1, 2] and strategies for linkage to cargo. Cargo can be virtually any biomolecule, ranging from large protein complexes [3] to small molecules to siRNA and other nucleic acids [4]. Their promise for therapeutics is high and more than 25 clinical trials are underway [5–7], but progress has been lacking due to a number of specific challenges including cargo coupling strategies, lack of cell specificity and poor endosomal escape.

It has long been noted that high doses of CPP-cargo fusions are required to generate measurable quantities in the cytoplasm as perhaps less than 1% of delivered cargo reaches the cytoplasm [8], and the dawning realization is that the vast majority CPPs get trapped in endosomes and targeted for degradation rather than released into the cytoplasm. The mechanism of entry is unclear, though receptor-mediated endocytosis is the most likely general mechanism [9, 10] with heparin sulfate proteoglycans as CPP receptors [11]. Perhaps high affinities of CPPs for their receptors are the reason why entrapment occurs.

Endosomal escape (also referred to as ‘endosomolysis’) remains a significant barrier to the adoption of CPP therapies and other applications. Several strategies have been developed to address this shortcoming. Use of endosomally cleavable peptides had a salutary effect on treatment of tumors in mice with a CPP-delivered toxin [12]. Other attempts include thiol [13] and photocleavable linkages [14]. Our prior report [15] was the first to use a specific, high-affinity, reversible noncovalent linkage to mediate cargo attachment to CPP moiety, thus overcoming the endosomal escape problem in a novel way.

Our prototype CPP-adaptor, TAT-CaM, consists of the CPP moiety of TAT [16] fused to human calmodulin (CaM). Calmodulin is a calcium biosensor that folds into a dumbbell-shaped conformation in the presence of Ca^2+^ [17–20], closing around a 17-residue calmodulin binding site (CBS) on target proteins. Binding of CaM to CBS motifs is around 1 nM in affinity in the presence of calcium but negligible in its absence [21]. Cargo proteins were expressed with a canonical CBS at the N-terminus. Cargos and CPP-adaptors bind spontaneously and rapidly in the presence of Ca^2+^. However, most mammalian cells maintain a low resting concentration of cytoplasmic calcium, typically ~ 100nM, ~20,000x less than extracellular concentrations [22] and endocytosed Ca^2+^ is rapidly released from endosomes [23]. Thus, cargos are released within minutes of entry even though the CPP-adaptor remains entrapped in the endosome. Importantly, significant release from the endosome was achieved at 1 μM, 10-100-fold less covalently linked CPP-cargos [8].

Thus, our system presents a solution to the endosomal escape problem through a penetration-then-release mechanism. In the present study, we sought to assay the generalizability of our scheme by using other CPP moieties SAP and SAP(E) [24, 25] and alternative EF hand proteins CALML3 [26] and troponin [27] as adaptors. All constructs delivered their cargos rapidly and with high efficiency, achieving cytoplasmic distribution within 1 hour, likely much faster.

We also sought to adapt our CPP-adaptor/cargo complexes to deliver to subcellular destinations. CPP-mediated delivery of cargo to places other than the cytoplasm has long been a goal of developing therapeutics as there are many sites of action [28]. By addition of localization signals to cargo proteins, delivery to the nucleus, peroxisomes and endoplasmic reticulum was achieved.

Lastly, our method holds the prospect to become a high-efficiency alternative to transfection. By far the most common method to manipulate mammalian cells is transfection. While tremendous efforts have been made to improve transfection efficiencies, they remain disappointing and many cell types are resistant to transfection. In our previous report [15] we noted that all cells in the populations to which we had delivered cargo received it, i.e. there was 100% efficiency. Hypothesizing that whatever barriers transfection encounters, they are not germane to CPP-mediated delivery, our CPP-adaptors were successfully used to deliver cargo to myotubes, suggesting that our adaptor system may be a useful alternative to liposome-mediated transfection, capable of delivering cargo of choice to a differentiated tissue structure.

## Materials and methods

### Strains and cell lines

*E*. *coli* strains NovaBlue (EMD Millipore, USA) and BL21(DE3)pLysS (Thermo Fisher, USA) were used, respectively, to propagate plasmids and express proteins. BHK21 (#CCL-10) and C2C12 mouse myoblasts (#CRL-1772) cells were purchased from ATCC. BHK was cultured in Dulbecco’s Modified Eagles’ Medium supplemented with 10% fetal bovine serum, 4 mM glutamine, and 4.5 g/L glucose. Myoblasts were cultured as described [29].

### Overexpression, purification and labeling

Plasmids encoding TAT-CaM and CBS-myoglobin have been previously described [16]. Genes encoding SAP-CaM, SAP(E)-CaM, TAT-Troponin (TAT-Tropo), TAT-calmodulin-like protein 3 (TAT-CALML3) were synthesized and cloned (Genewiz, South Plainfield, NJ) into *NdeI* and *BamHI* sites in pET19b (EMD Millipore, USA). Troponin inhibitory peptide-myoglobin (TIP-myo)[26] and CBS-myoglobins with C-terminal consensus subcellular localization signals for the nucleus (NLS), endoplasmic reticulum (KDEL) and peroxisomes (SKL) and CBS-α-tubulin were synthesized and cloned into *BamHI* and *HindIII* sites on pCAL-N-FLAG (Agilent Technologies, CA, USA), which encodes a calmodulin binding peptide and FLAG epitope N-terminal to the *BamHI* site. All synthetic genes were codon-optimized for expression in *E*. *coli*. Plasmids are listed in Table 1.

**Table 1:** Plasmids used in this study.

Identifier	Descriptor	Parent Vector	Decription	Relevant GenBank Accession #s
pJM996	TAT-CaM	pET19b	N-His-TAT-CaM	NP_001734.1
pJM1000	TAT-CaM 2.0	pET19b	N-His-TAT-CaM without GS C-terminal to TAT	NP_001734.1
pJM995	CBS-myo	pCAL-N-FLAG	N-CBS-myoglobin	AAA59595.1
pJM1002	SAP-CaM	pET19b	N-His-SAP-CaM	NP_001734.1
pJM1003	SAP(E)-CaM	pET19b	N-His-SAP(E)-CaM	NP_001734.1
pJM1001	TAT-CALML3	pET19b	N-His-TAT-calmodulin like protein 3	NP_005176.1
pJM1020	TAT-troponin	pET19b	N-His-TAT-troponin	NP_003270.1
pJM1021	TIP-myo	pET19b	N-His-CBS-troponin inhibitory peptide-myoglobin	AAA59595.1
pJM985	Myo-NLS	pCAL-N-FLAG	N-CBS-myoglobin-NLS	AAA59595.1
pJM1022	Myo-KDEL	pCAL-N-FLAG	N-CBS-myoglobin-KDEL	AAA59595.1
pJM987	Myo-SKL	pCAL-N-FLAG	N-CBS-myoglobin-SKL	AAA59595.1
pJM1023	CBS-tubulin	pCAL-N-FLAG	N-CBS-tubulin α1A (mouse)	NP_035783.1

Plasmids used in this study. Accession numbers are for the naturally occurring gene from which codon-optimized sequences were generated.

Proteins were expressed essentially as described [30]. Briefly, plasmids were transformed into BL21(DE3)pLysS. Overnight cultures were subcultured into 1L Luria-Bertani Broth and grown with vigorous shaking at 30°C. At OD_600_ ~0.4 cells were induced with 0.2 mM IPTG and growth continued for four hours. Cells were harvested and frozen at -80°C.

Purification was also performed essentially as described [15], with CPP constructs purified via immobilized metal affinity chromatography and CBS-cargo constructs purified using a Calmodulin Sepharose column (GE Life Sciences, Pittsburgh, PA, USA). Proteins were exchanged into 10 mM HEPES, 150 mM NaCl, 10% glycerol for biotinylation or fluorescence labeling, both of which were accomplished by amine crosslinking. For biotinylation of CPP constructs used in optical biosensing experiments, NHS-LC-LC biotin was crosslinked per the manufacturer’s protocol (ThermoFisher, USA). For confocal microscopy experiments, DyLight 550 was similarly crosslinked to cargo proteins or TAT-CaM and dye removal columns were used to remove unreacted dye (ThermoFisher). All proteins were exchanged into binding buffer (10 mM Tris, 150 mM NaCl, 10% glycerol, 1 mM CaCl_2_ pH 7.4) by passage over a gel filtration column prior to analysis.

### Optical biosensing

All biolayer interferometry measurements were carried out on a FortéBio (Menlo Park, CA) Octet QK biosensor with streptavidin sensors at 25°C on 96-well opaque plates. All volumes were 200 μl. Biotinylated CPP constructs were loaded for 300s after which sensors were moved to binding buffer only and a baseline was established. Association and dissociation phases were 300s each. Raw data were reference subtracted against the signal from a ligand-loaded sensor against buffer only and were then fit using a global one-state association-then-dissociation model with GraphPad Prism 5.03, from which kinetic and affinity constants were determined. Nonspecific binding was measured with respect to the response of 1 μM analyte protein against a sensor without ligand and was found to be negligible in all cases. Observation of rapid dissociation in the absence of calcium was accomplished by movement of the sensors into wells containing binding buffer with 10 mM EDTA and measuring nm shift for 300 s.

### Cell penetration assays

1 μM each of TAT-CaM and DyLight 550-labelled cargo protein in buffer containing 1 mM CaCl_2_ were added to subconfluent BHK21 cells and incubated for 1 hour, after which cells were washed three times in phosphate buffered saline with 1 mM CaCl_2_. The cytoplasm was labeled by treatment with CytoTracker 488 Dye (Molecular Probes, USA). Cells were also labelled with NucBlue Live (Thermo Fisher, USA) and transferred to media containing 25 mM HEPES, pH 7.4 for imaging. Cells were immediately imaged on an inverted Zeiss LSM700 Confocal Microscope equipped with a 40x EC Plan-Neofluar objective (NA = 1.3). Pinholes for each fluorophore were set at 1.0 Airy Units (29 microns), and SP 490 and LP 615 filters were used to acquire the NucBlue (blue channel) and DyLight 550 (Red Channel) signals, respectively.

Z-stacks for both TaT-CaM-treated and untreated cells were set by using the NucBlue staining of the nucleus as a reference (typical Z-stacks ranged from 6.0–10.0 microns). Both treated (CPP-adaptor plus cargo) and untreated (cargo only) cells were imaged at identical gain settings, set at sub-saturation levels on cells treated with TaT-CaM in the red emission (DyLight 550 fluorescence). Both treated and untreated cells were imaged at an identical laser output level (2.0%, 555 nm laser), identical pixel dwell time of 3.15 microseconds, and 2x line averaging.

For analysis, images were rendered using the Orthogonal View in Zen Blue (Zeiss, Germany) software. Using the diameter of the nucleus as a landmark, the Z-plane chosen for analysis corresponded to approximately the mid-point depth of the nucleus. Finally, the DyLight 550 signal was analyzed separately and merged with NucBlue. For subcellular localization experiments, appropriate compartments (e.g., peroxisome, endoplasmic reticulum) were labeled with CellLight Peroxisome-GFP, BacMaM 2.0 (Molecular Probes)[31], or ERTracker Dye (Molecular Probes), respectively according to the manufacturers’ protocols.

Experiments to confirm TAT-CaM localization essentially as described above except that the TAT-CaM was labelled with DyLight 550, the cargo, CBS-myo was unlabelled and the complexes were added at equimolar ratios of 10 nM and 100 nM in separate experiments.

For delivery of protein cargo to myotubes, we first cultured C2C12 myoblasts on Matrigel-coated Ibidi plastic culture dishes (Ibidi, USA), under standard media conditions for growth and differentiation [29]. Myotubes were differentiated for three days, and then protein cargos and labeling of myotubes, as well as imaging were performed as described above.

## Results & discussion

A prior study demonstrated the utility of our CPP-adaptor strategy for delivery of molecular cargo to the interiors of cells with TAT-CaM and several different model cargos and several different cell lines [16]. All cargos were widely distributed in the cytoplasm and three different cell types were readily penetrated. The present results show extended utility of noncovalent coupling in the use of different CPP moieties, the differential fates of CPP-adaptors and cargos and effective distribution using subcellular localization signals.

**Fig 1:**
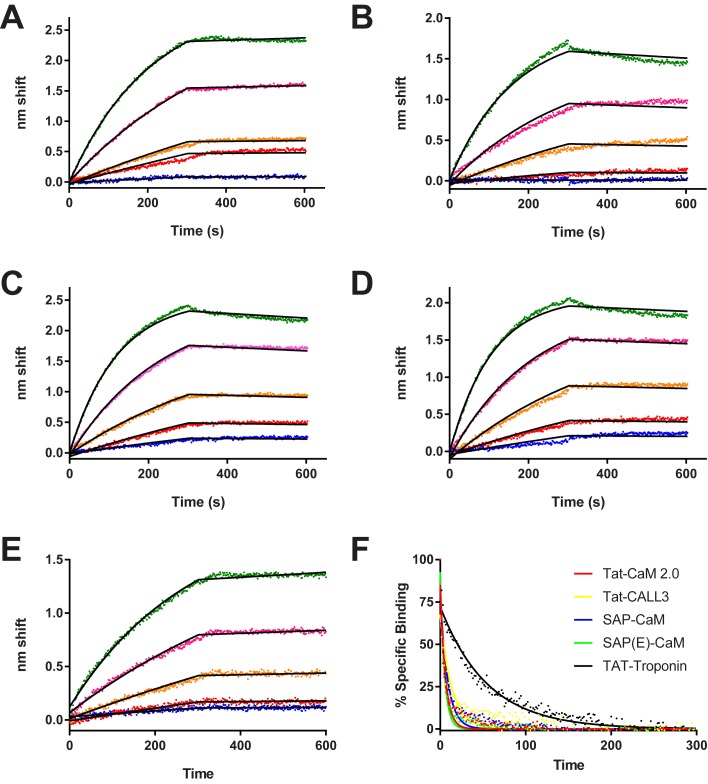
Biolayer interferometry analysis of CPP-adaptor-cargo binding. Association (0-300s) and dissociation (300-600s) phases are shown for A, TAT-CaM; B, TAT-CALL3; C, SAP-CaM; D, SAP(E)-CaM and E, TAT-Tropo binding to CBS-myoglobin (A-D) or TIP-myoglogbin. Analyte concentrations were 1000 nM (green), 500 nM (magenta), 250 nM (orange), 125 nM (red), and 63 nM (blue). Fits are shown to a single-state global model for which the constants are shown in Table 1. F, EDTA dissociation phases for 1000 nM analyte samples moved into EDTA after dissociation phase. Binding normalized to % specific binding to eliminate differences in amplitude, allowing direct comparisons.

The generalizability of the use of EF hand proteins and their cognate binding targets was open to question. We designed a second generation TAT-CaM, TAT-CaM, 2.0, to remove an extraneous pair of residues within the fusion tag that were the result of an unneeded restriction site engineered into the original synthetic gene; it is otherwise identical. We used calmodulin-like protein 3 (CALML3) and troponin as alternate EF hand proteins as well as SAP and SAP(E) as alternate CPP moieties of differing characteristics. As shown in Fig 1A–1E, CPP-adaptors bound their cargos with high affinities in the presence of calcium, as expected. The cargo protein was CBS-myo for all CPP-adaptors save TAT-Troponin, for which the cargo was troponin inhibitory peptide-myoglobin, TIP-myo.

Affinities ranged from 13–30 nM. k_off_ and K_D_ were indeterminate for TAT-CaM 2.0 and TAT-troponin binding to their cargos as anomalies in the early parts of the dissociation phases made determination of their very low off rates impossible; suffice it to say their k_off_s are very low and the affinity of TAT-CaM 2.0 and TAT-troponin for their cargos is very high in the presence of calcium.

The identities of the cell-penetrating sequence and EF hand proteins did not significantly affect binding kinetics as SAP and SAP(E) were indistinguishable from TAT-CaM 2.0, which itself is indistinguishable from TAT-CaM [16]. Other adaptors using alternate EF hand proteins, TAT-CALL3 and TAT-troponin, bound their targets with high affinity. On rate constants for all constructs are on the order of 10^3^ M^-1^ s^-1^ and off rate constants are ~10^−4^ s^-1^ or slower, in good agreement with our prior measurements for TAT-CaM and several model cargos [16] as well as wild-type calmodulin [22]. Kinetic parameters are shown in Table 2.

**Table 2:** Kinetic and affinity constants for CPP-adaptor binding to CBS-myoglobin (or TIP-myoglobin).

Constant	TAT-CaM 2.0	TAT-CALML3	SAP-CaM	SAP(E)-CaM	TAT-Troponin
k_on_ (M^-1^s^-1^)	4900	6100	8300	9400	3700
k_off_ (s^-1^)	ND	1.8 x 10^−4^	1.7 x 10^−4^	1.3 x 10^−4^	ND
K_D_ (nM)	ND	29.8	20.6	13.3	ND
k_off_ (EDTA) (s^-1^)	0.17	0.07	0.11	0.20	0.018

Kinetic and affinity constants as determined from global fits of sensorgrams shown in Fig 1. ND, not determined.

All CPP-CaM-cargo complexes exhibited very fast dissociation (~10^−1^ s^-1^) when exposed to EDTA (Fig 1F), as expected and previously shown of calmodulin and its regulated proteins in upon the removal of calcium [15, 21, 32, 33]. Interestingly TAT-Tropo exhibited slower, but still rapid dissociation (1.8 x 10^−2^ s^-1^). This may be due to the conformational shift of troponin C that accompanies Ca^2+^ dissociation being slow compared to calmodulin [26], though it may also represent some other difference in CPP-adaptor/cargo pair. However, regardless of particular CPP, adaptor or binding sequence, all CPP-adaptor/cargo pairs bound with high affinity in the presence of calcium and low affinity in its absence, demonstrating that our overall design of high-affinity, reversible cargo coupling is valid.

**Fig 2:**
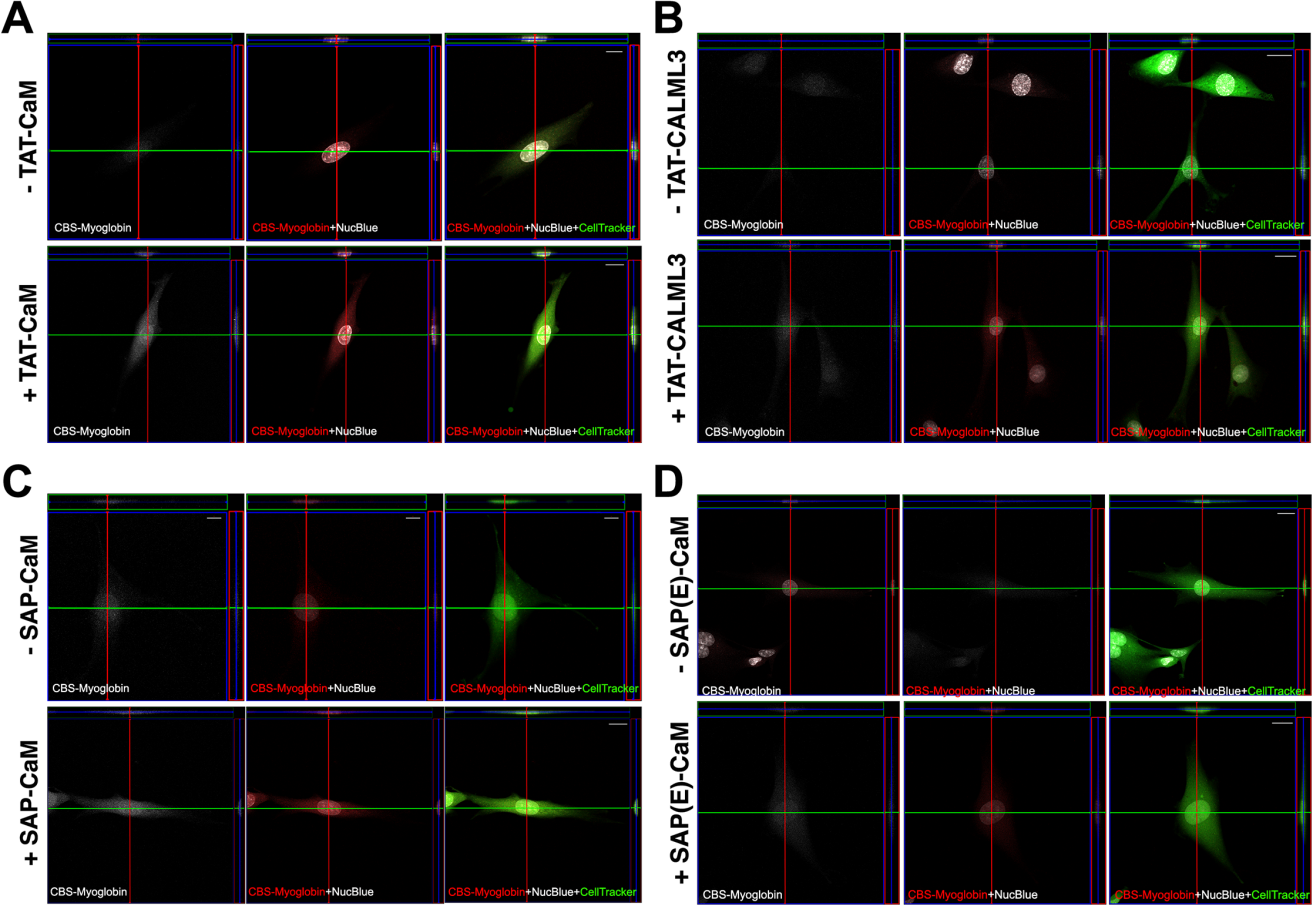
Cell penetration assay for a variety of CPPs and adaptor proteins for delivery of myoglobin to BHK cells. Each panel are images from experiments with different CPP-adaptors: A, TAT-CaM 2.0; B, CALML3; C, SAP-CaM; D, SAP(E)-CaM. BHK cells were treated for 1 h with DyLight 550 fluorescently labeled CBS-myo (rendered as white in left panels, red in center and right panels), in either the absence or presence of CPP-adaptor, washed and imaged live. Center images are optical sections set at a similar depth of the nucleus (NucBlue staining, white, center and right panels), as determined by position within the Z-stack. Orthogonal projections are shown at the right (boxed in red) and top (boxed in green) sides of each panel. Cytoplasmic compartments in live cells were visualized using CellTracker Green CMFDA dye (green in right panels). Comparison of CPP-adaptor-treated versus untreated cells indicates that in all cases, myoglobin was delivered and localized primarily to the cytoplasm. Scale bars in all panels, 20 μm.

Cell penetration experiments likewise demonstrated effective delivery and cytoplasmic distribution of cargo proteins in BHK cells (Fig 2). In the presence of an equimolar (1 μM) concentration of the CPP-adaptor, all fluorescently labelled cargo proteins colocalized with cytoplasmic tracking dye. When CPP-adaptors were absent, fluorescence was negligible, which was underscored by its absence at the same cytoplasmic depth as evidenced by the orthogonal projections, i.e. the fluorescence present in the absence of CPP-adaptors is due to nonspecific adherence to the outside of the cell. Attempts to characterize the kinetics of penetration failed as there was no condition under which we could observe the cargo in which it was not widely distributed throughout the cytoplasm, i.e. the time it took from treatment to image acquisition was longer than penetration and release. Rapid cytoplasmic distribution is consistent with rapid loss of endocytosed Ca^2+^ coincident with acidification of endosomes [23].

**Fig 3:**
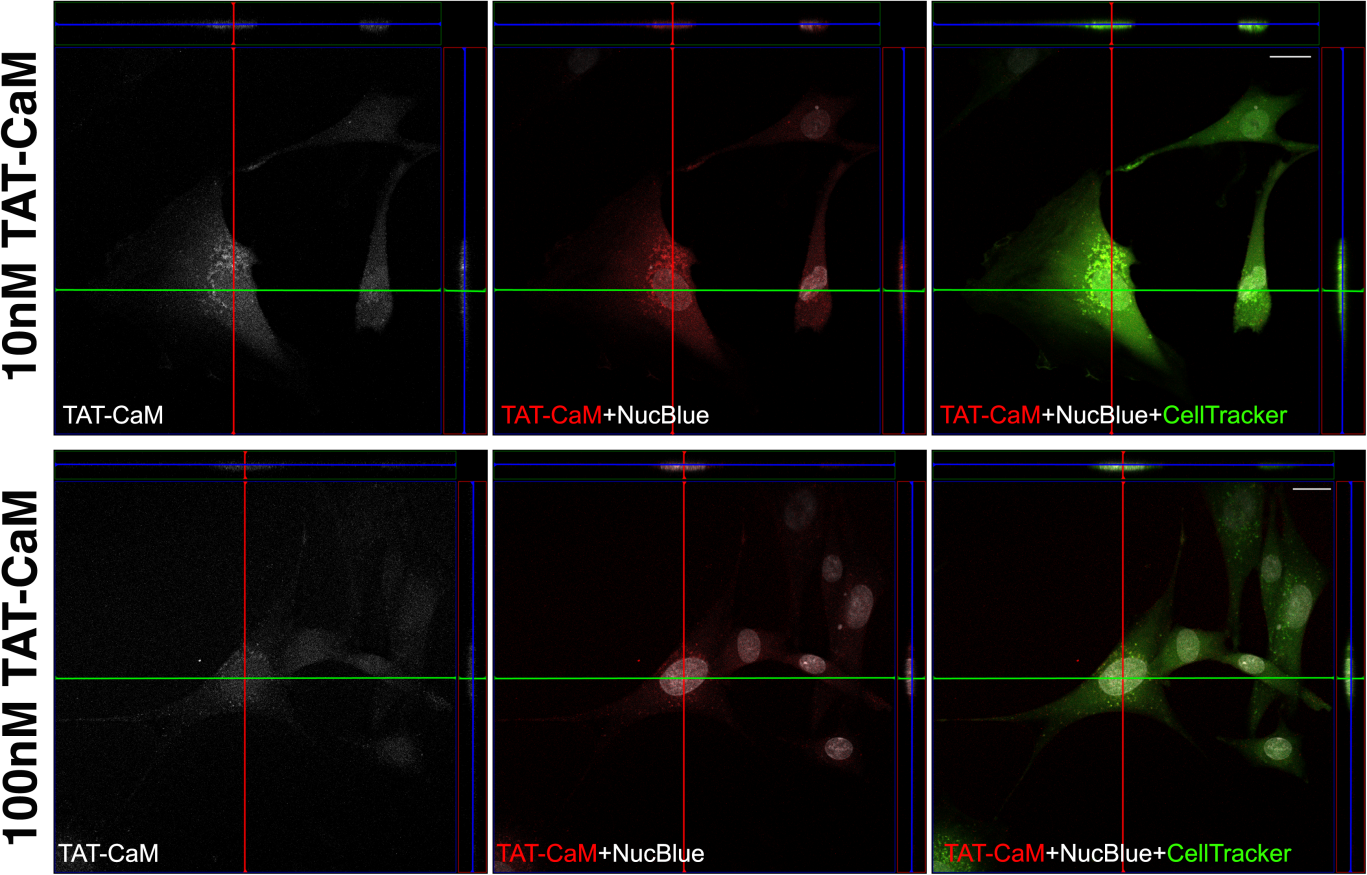
TAT-CaM localization. Cell penetration assay performed with complexes of fluorescently labelled TAT-CaM and unlabeled myoglobin at 10 nM (top) and 100 nM (bottom). Fluorescence rendering is the same as Fig 1 (Dylight fluorescence is white in the left panels and red in the center and right panels).

What of TAT-CaM? Does it get into the cytoplasm or is it trapped in endosomes as other TAT constructs? To address whether our CPP-adaptor is fundamentally the same as other TAT moieties, a penetration experiment was conducted in which TAT-CaM was labelled and CBS-myo was unlabeled (Fig 3A). As expected, TAT-CaM exhibited a punctate distribution consistent with endosomal localization. To confirm the result, a similar assay was conducted with unlabeled proteins, after which cells were fixed and then exposed to anti-calmodulin and a fluorescently labelled secondary antibody (S1 Fig). The punctate distribution of TAT-CaM was pronounced, leading to the conclusion that indeed, our TAT-CaM remains trapped in the endosomes but releases cargo for subsequent escape to the cytoplasm.

**Fig 4:**
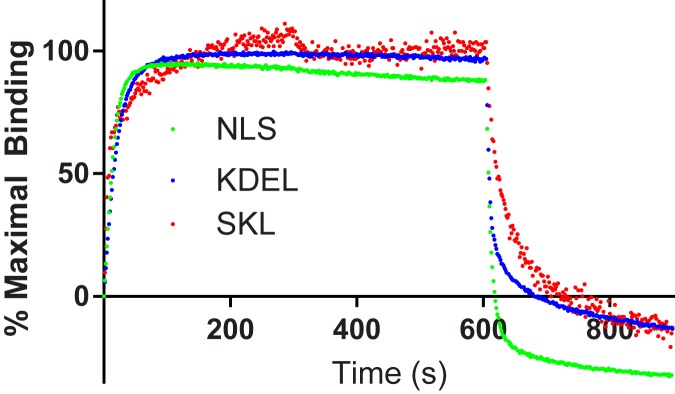
Biolayer interferometry analysis of subcellular localization constructs. TAT-CaM was used as ligand and analytes were CBS-myoglobin-NLS (green), CBS-myoglobin-KDEL (blue), CBS-myoglobin-SKL (red). Phases, analyte concentrations and fits are the same as in Fig 1.

CPP-mediated delivery of cargos to subcellular compartments has long been desired but has similarly been stymied by the endosomal escape problem [11]. Having demonstrated the advantages of our noncovalent linking strategy, we also sought to use recombinant cargos with subcellular localization signals. CBS-myo constructs with C-terminal subcellular localization signals were expressed, purified and analyzed with respect to binding, penetration and localization. Localization signals examined were nuclear (SV40 large T antigen signal, PKKKRKV), peroxisomal (SKL) and endoplasmic reticulum (KDEL)[34]. As expected (Fig 4), all constructs exhibited high affinity, fast-on, slow-off binding to TAT-CaM in the presence of calcium and rapid dissociation in the presence of EDTA. Full characterizations were not done and amplitude was normalized to percent maximal binding because the binding experiments were performed with different ligand preparations. Signal less than zero in the EDTA phase is likely a result of association and EDTA-induced dissociation phases so rapid that a significant amount occurred during transitions of the sensor from baseline to association and from dissociation to EDTA dissociation; the instrument takes a reading every 1.6 seconds; rapid binding or dissociation can cause uncertainty as to where 0 nm shift is.

**Fig 5:**
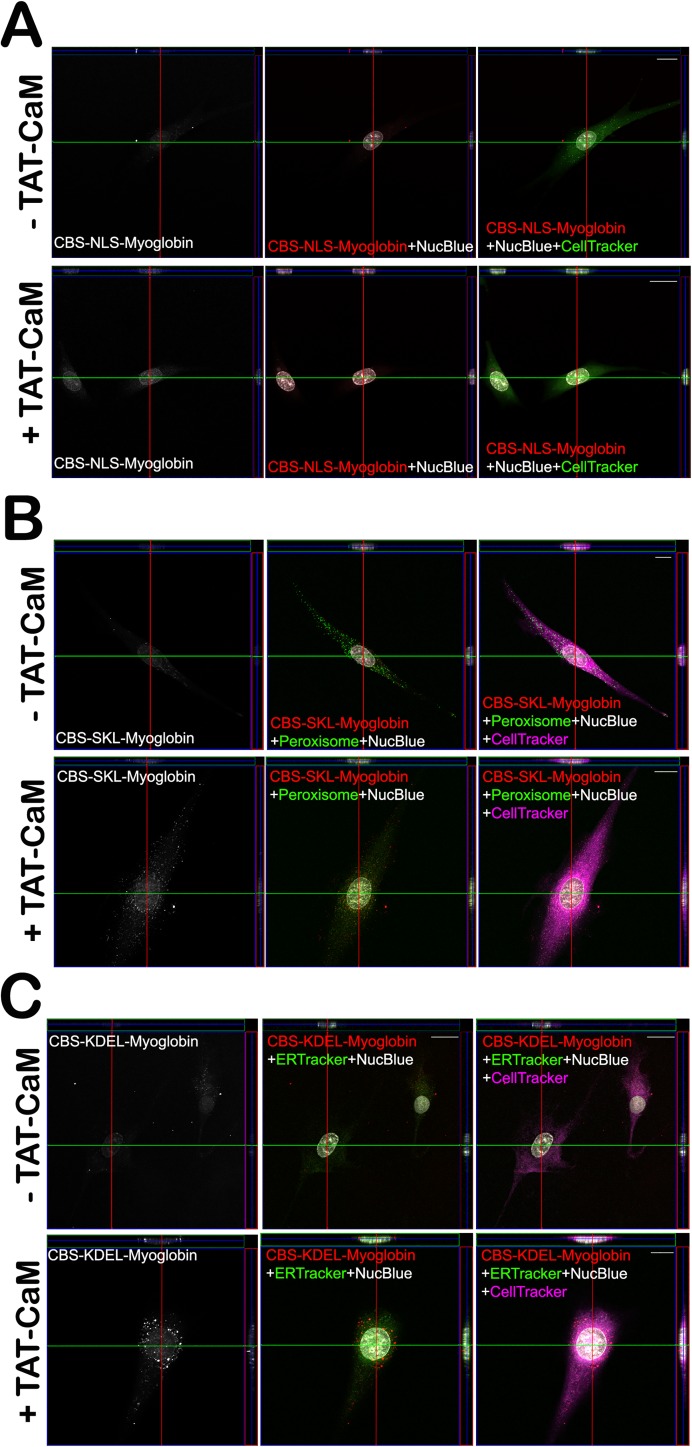
Cell penetration assay for subcellular localization. Each panel are images from a different cargo protein: A, CBS-myo-NLS (nuclear localization signal); B, CBS-myo-SKL (peroxisome signal); C, CBS-myo-KDEL (endoplasmic reticulum signal). Cells were treated and images are rendered the same as Fig 2 except that for panels B and C, CellTracker (cytoplasmic marker) is purple and either peroxisome (B) or ER (C) markers are rendered in green. Comparison of CPP-adaptor-treated versus untreated cells indicates that in all cases, myoglobin was delivered and localized to the appropriate subcellular compartment. Scale bars in all panels, 20 μm.

All localization signals exhibited delivery to intended destinations (Fig 5). Addition of either nuclear localization sequence, KDEL, or SKL tags resulted in delivery of cargo protein to either the nuclei, ER, or the peroxisomeas indicated by colocalization with the respective compartment labels. To our knowledge, our CPP-adaptor system is first CPP-mediated delivery method to readily achieve efficient penetration and disparate distribution. That it is relatively simple, utilizing the well-studied TAT sequence, reversible high-affinity binding and consensus localization signals strongly suggest its general utility as a research tool and hint at its promise for therapeutic delivery.

**Fig 6:**
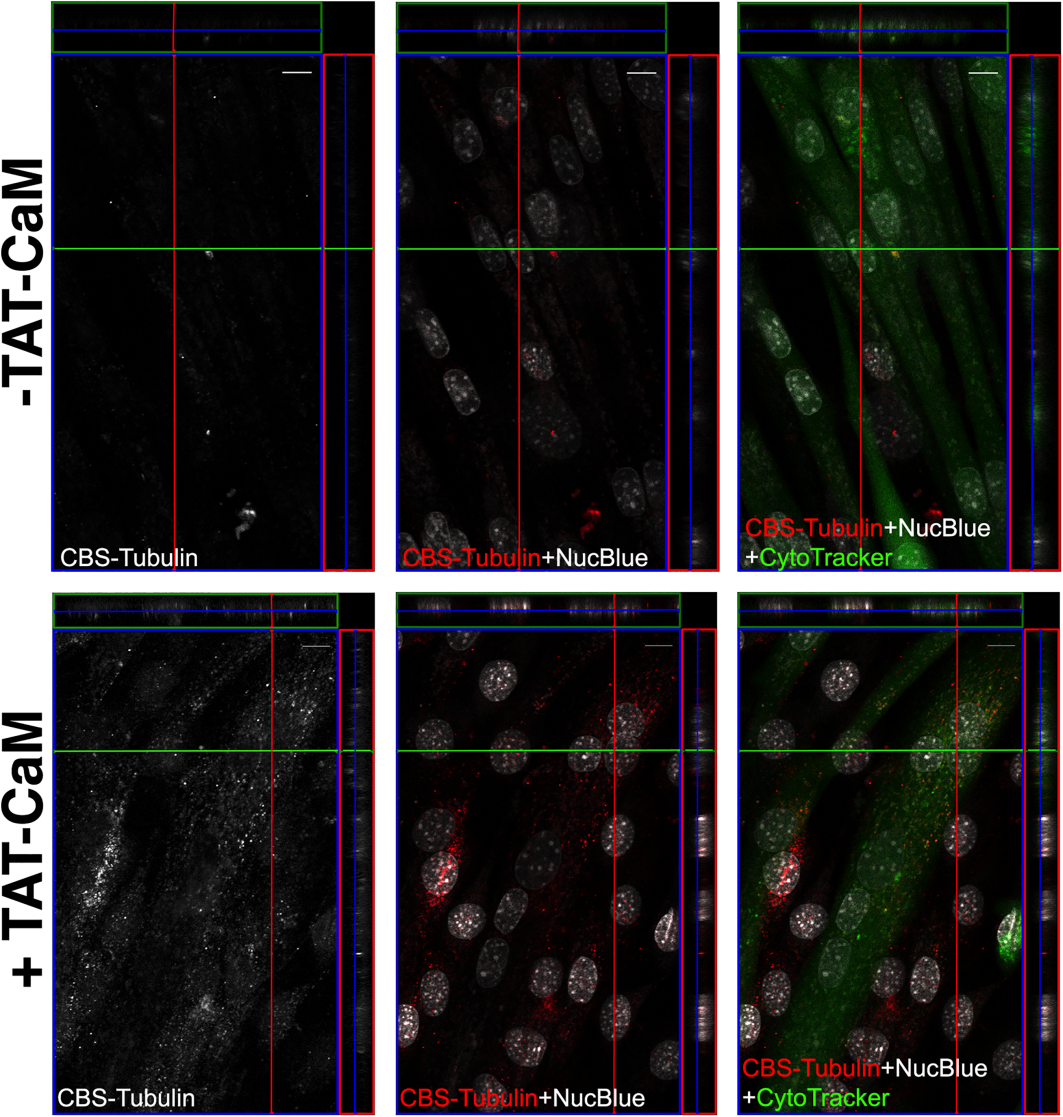
Cell penetration in myotubes. DyLight 550-labelled tubulin was used as cargo for TAT-CaM-mediated delivery to myotubes. Colors are rendered as in Fig 2.

Conventional liposomal transfection protocols require recipient cells to be actively dividing. However, differentiated cell structures, such as cultured mouse myotubes, are notoriously difficult to transfect using nonviral or non-electroporation based methods, as these fully differentiated cells have largely exited the cell cycle [35]. Our protein delivery method can likely overcome this barrier. To address the facility of delivery of cargo to such a cell line, we assayed fused, differentiated C2C12 myotubes for delivery of CBS-α-tubulin. As expected, cargo tubulin was delivered in the presence, but not the absence of TAT-CaM, and its distribution was found throughout the cytoplasm (Fig 6). We are tremendously excited by this finding, as we have successfully delivered a cargo of choice in a safe, non-viral method, to a fully differentiated tissue structure, under largely normal tissue culture conditions.

## Supporting information

S1 Fig Immunohistological confirmation of TAT-CaM localization. BHK cells were treated with TaT-CaM complexed with CBS-Myo (100 nM each) for 15 min. Cells were washed in PBS 3 times and then fixed with 4% PFA and 0.1% Triton-X-100 with rocking at room temperature for 20 min, then blocked with PBS/5% BSA for an hour. The fixed cells were probed overnight at 4^°^C with a 1:1000 dilution of anti-calmodulin (Thermo Fisher). Localization was observed using a 1:200 dilution Alexa 488-labelled goat anti-rabbit Alexa 488.
(PPTX)
