# Supplementary material for: A versatile cell-penetrating peptide-adaptor system for efficient delivery of molecular cargos to subcellular destinations
Source: PLoS One. 2017 May 26;12(5):e0178648. doi: 10.1371/journal.pone.0178648 (PMC5446193; doi:10.1371/journal.pone.0178648)
Supplement: S1 Fig — BHK cells were treated with TaT-CaM complexed with CBS-Myo (100 nM each) for 15 min. Cells were washed in PBS 3 times and then fixed with 4% PFA and 0.1% Triton-X-100 with rocking at room temperature for 20 min, then blocked with PBS/5% BSA for an hour. The fixed cells were probed overnight at 4°C with a 1:1000 dilution of anti-calmodulin (Thermo Fisher). Localization was observed using a 1:200 dilution Alexa 488-labelled goat anti-rabbit Alexa 488. (PPTX) [file pone.0178648.s001.pptx]

## Slide 1
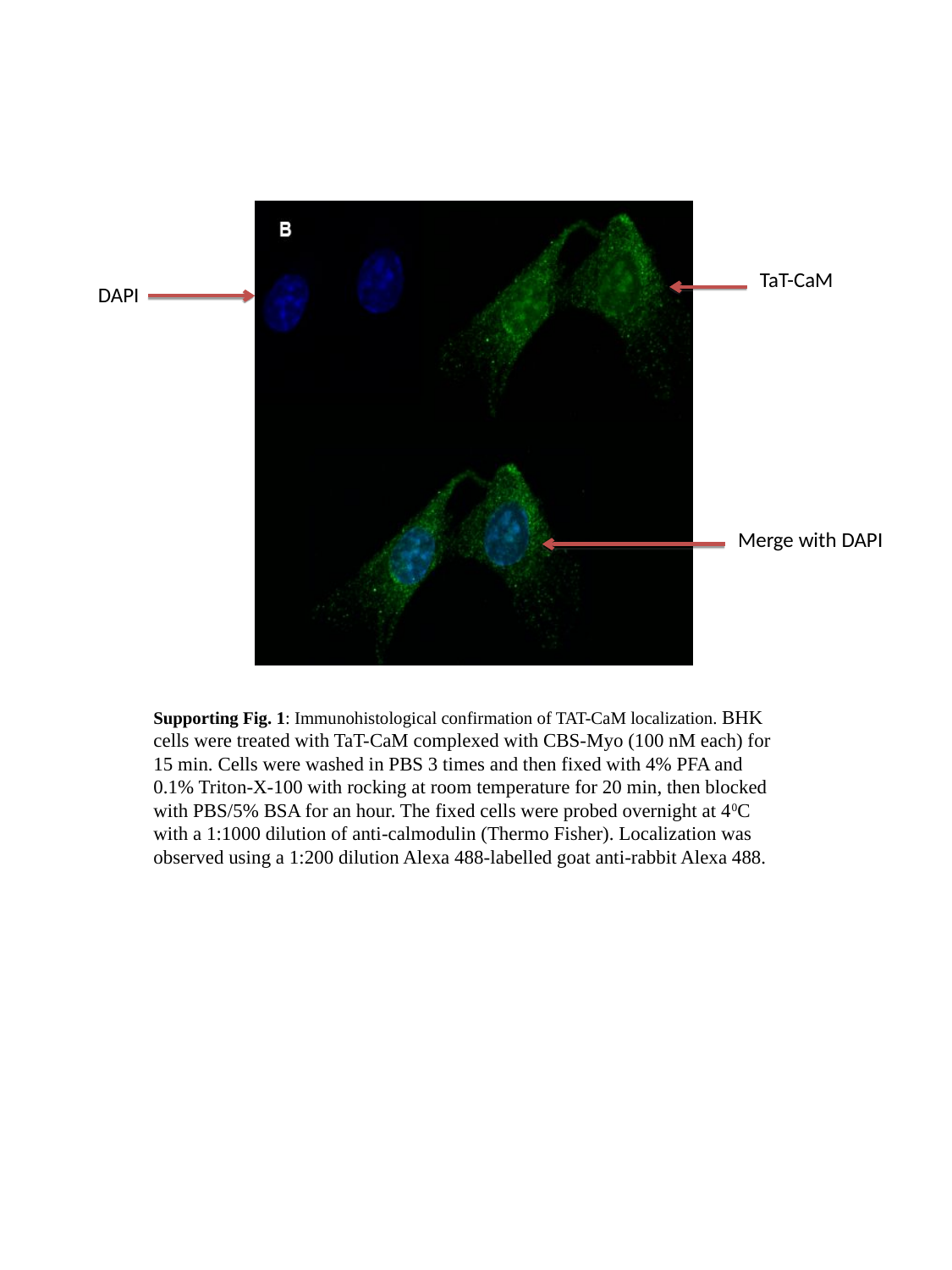

TaT-CaM
DAPI
Merge with DAPI
Supporting Fig. 1: Immunohistological confirmation of TAT-CaM localization. BHK cells were treated with TaT-CaM complexed with CBS-Myo (100 nM each) for 15 min. Cells were washed in PBS 3 times and then fixed with 4% PFA and 0.1% Triton-X-100 with rocking at room temperature for 20 min, then blocked with PBS/5% BSA for an hour. The fixed cells were probed overnight at 40C with a 1:1000 dilution of anti-calmodulin (Thermo Fisher). Localization was observed using a 1:200 dilution Alexa 488-labelled goat anti-rabbit Alexa 488.
